# Obesity and BMI Cut Points for Associated Comorbidities: Electronic Health Record Study

**DOI:** 10.2196/24017

**Published:** 2021-08-09

**Authors:** Natalie Liu, Jen Birstler, Manasa Venkatesh, Lawrence Hanrahan, Guanhua Chen, Luke Funk

**Affiliations:** 1 Department of Surgery University of Wisconsin-Madison Madison, WI United States; 2 Department of Biostatistics and Medical Informatics University of Wisconsin-Madison Madison, WI United States; 3 Department of Family Medicine and Community Health University of Wisconsin-Madison Madison, WI United States; 4 Department of Surgery William S. Middleton Memorial VA Madison, WI United States

**Keywords:** obesity, body mass index (BMI), risk factors, screening, health services, chronic disease

## Abstract

**Background:**

Studies have found associations between increasing BMIs and the development of various chronic health conditions. The BMI cut points, or thresholds beyond which comorbidity incidence can be accurately detected, are unknown.

**Objective:**

The aim of this study is to identify whether BMI cut points exist for 11 obesity-related comorbidities.

**Methods:**

US adults aged 18-75 years who had ≥3 health care visits at an academic medical center from 2008 to 2016 were identified from eHealth records. Pregnant patients, patients with cancer, and patients who had undergone bariatric surgery were excluded. Quantile regression, with BMI as the outcome, was used to evaluate the associations between BMI and disease incidence. A comorbidity was determined to have a cut point if the area under the receiver operating curve was >0.6. The cut point was defined as the BMI value that maximized the Youden index.

**Results:**

We included 243,332 patients in the study cohort. The mean age and BMI were 46.8 (SD 15.3) years and 29.1 kg/m^2^, respectively. We found statistically significant associations between increasing BMIs and the incidence of all comorbidities except anxiety and cerebrovascular disease. Cut points were identified for hyperlipidemia (27.1 kg/m^2^), coronary artery disease (27.7 kg/m^2^), hypertension (28.4 kg/m^2^), osteoarthritis (28.7 kg/m^2^), obstructive sleep apnea (30.1 kg/m^2^), and type 2 diabetes (30.9 kg/m^2^).

**Conclusions:**

The BMI cut points that accurately predicted the risks of developing 6 obesity-related comorbidities occurred when patients were overweight or barely met the criteria for class 1 obesity. Further studies using national, longitudinal data are needed to determine whether screening guidelines for appropriate comorbidities may need to be revised.

## Introduction

### Background

Obesity (BMI ≥30.0 kg/m^2^) is a global public health problem. The highest rates of obesity occur in the United States, where over one-third of adults have obesity [1]. In 1998, the World Health Organization created international standardized BMI classifications for adults who are overweight and have obesity based on risks of obesity-related diseases for European adults [2]. These classifications were based on the risks of obesity-related diseases in European adults with varied BMI values [3]. On the basis of these classifications, overweight and obesity were defined as having a BMI between 25.0 and 29.9 kg/m^2^ and a BMI≥30.0 kg/m^2^, respectively. However, studies have demonstrated that the risks of obesity-related comorbidities differ based on sex and race or ethnicity. Female Asian patients have been shown to develop comorbidities at lower BMIs, suggesting that BMI thresholds for overweight and obesity should be lower for these groups [2,4-7].

### Study Significance

Obesity is associated with numerous comorbidities, including hypertension, hyperlipidemia, type 2 diabetes mellitus (T2DM), and coronary artery disease (CAD) [8-10]. The cross-sectional study by Pantalone et al [8], which used electronic health record (EHR) data, showed that patients with higher BMIs had a higher prevalence of T2DM, hypertension, and CAD. However, studies have not addressed whether specific BMI cut points exist for US adults. BMI cut points are defined as the thresholds beyond which disease incidence can be accurately detected. In addition, no studies have evaluated cut points by using EHR data that provide patient-level information for large, multiethnic cohorts. Studies have concluded that it is feasible to use EHR analysis to study chronic diseases such as obesity, diabetes, and hypertension [11,12].

### Objective

The objective of this study is to examine EHR data from a large health care system in the United States to determine whether BMI cut points exist for 11 common comorbidities associated with obesity and being overweight. We also evaluate whether cut points varied with sex and race or ethnicity. We hypothesize that most cut points would occur in the class 1 obesity category.

## Methods

### Data Source

We used data from the University of Wisconsin Hospital and Clinics EHR over a 10-year period (June 1, 2008, to December 31, 2018). All patient data and analyses were stored on a secure server managed through the University of Wisconsin Health Information Services and the Institute for Clinical and Translational Research. The Epic Clarity Database was used as the data source for all patients. This study was approved by the University of Wisconsin Minimal Risk institutional review board (protocol #2017-0443), and the need for informed consent was waived. We followed the Strengthening the Reporting of Observational Studies in Epidemiology guidelines within the Enhancing the Quality and Transparency of Health Research network in the methodology and reporting of this study (Multimedia Appendix 1 contains the full Strengthening the Reporting of Observational Studies in Epidemiology checklist) [13].

### Data Validation and Cleaning

All recorded heights and weights in the EHR were cleaned to reduce the inclusion of incorrect heights and weights because of errors in data entry. Similar to our previous study using EHR data, we used the methodology proposed by Cheng et al [14] to remove biologically implausible heights and weights [15]. All heights >90 inches, <44 inches, and >1 SD from the mean height when SD was >2.5% of the mean were removed. All weights >1000 pounds, <55 pounds, >70% of the range from the mean when the range ≥50 pounds, and >1 SD from the mean when the SD was >20% of the mean were removed. Missing height data were imputed with the most recent previous nonmissing valid height. Any remaining missing height was replaced with the most recent subsequent nonmissing valid height. BMI values were calculated using the valid heights and weights. No patients were excluded from the study because of the data cleaning process.

### Study Population

We included all patients between the ages of 18 and 75 years who had ≥3 in-person clinical visits over a minimum of 2 years documented in the EHR during the study period. All included patients had an *index visit* with a valid BMI measurement, another visit at least 1 year before the index visit, and an additional visit 1 year after their index visit. The minimum 1-year period between the index visit and the previous visit was used to identify patients who had each disease of interest versus those who did not. The 1-year period between the index visit and the subsequent visit was used to calculate 1-year incidence rates for patients who did not have the disease before the index visit but were later diagnosed with the disease. Patients with multiple intervals of ≥3 clinical visits had an interval selected at random.

Patients with a pregnancy or cancer diagnosis at any time before or during the study period were excluded using the International Classification of Disease (ICD)-9 and ICD-10 codes. Patients who had undergone bariatric surgery were identified from our institutional bariatric surgery registry and excluded.

### Study Variables

Baseline BMI (BMI at the index visit), age (at the index visit), sex (male or female), race or ethnicity (White, non-Hispanic; Black, non-Hispanic; Asian, non-Hispanic; Native American, non-Hispanic; Hispanic; or other or unspecified), insurance type (commercial or private insurance, Medicare, Medicaid, or other or unspecified), and smoking status (at the index visit; active smoker, former smoker, passive smoker [defined as an individual who has had exposure to tobacco smoke but has never smoked themselves], or nonsmoker) were identified from the EHR. Insurance type was defined as the insurance type used during or before the index visit.

Through a literature review, we identified 11 common obesity-related comorbidities that were included in this study: anxiety, CAD, cerebrovascular disease, chronic pain, depression, gastroesophageal reflux disease, hyperlipidemia, hypertension, obstructive sleep apnea (OSA), osteoarthritis, and T2DM [8,9,16,17]. Incident cases were defined as patients who did not have the disease before the index visit and subsequently developed the disease after the index visit. The 1-year incidence rates (defined per 100 person-years) were calculated based on the occurrence of an ICD-9 or ICD-10 code (Multimedia Appendix 2 contains the full list of ICD-9 and ICD-10 codes) during the 1-year period following the index visit for patients who did not have a diagnosis before the index visit. Prevalent cases were defined as patients who had a diagnosis of comorbidity at or before the index visit and identified using the occurrence of an ICD-9 or ICD-10 code during this time.

### Statistical Analysis

We used quantile regression with BMI as the outcome to identify differences in the median BMIs between incident cases of each comorbidity and those who did not develop each comorbidity. Two models were fit for each comorbidity to evaluate the associations between BMI and disease incidence—an unadjusted model with disease incidence as the only independent variable and an adjusted model accounting for baseline age, sex, race or ethnicity, and smoking status. We used quantile regression because we were unable to meet the assumptions of the linear model. Quantile regression also allowed for the evaluation of differences in BMI distributions among patients who developed each comorbidity versus those who did not, which is more informative than differences in single mean values [18]. The difference in median BMIs (the median BMI of incident cases minus the median BMI of patients who did not develop the disease) was the outcome of the quantile model.

We conducted cut point analyses with BMI as a screening test for the incidence of each obesity-related comorbidity. Sensitivity and specificity were calculated for continuous BMI values. A comorbidity had a BMI cut point if the area under the receiver operating curve (AUROC) was >0.6. We chose an AUROC>0.6 to ensure that cut points had significant diagnostic value. Although there is no gold standard method, other investigators have used AUROC thresholds that range from >0.5 to >0.7 to determine cut points [6]. For all comorbidities with an AUROC >0.6, the cut point was defined as the BMI value that maximized the Youden index (sensitivity+specificity-1). BMI cut points were also calculated by sex and race or ethnicity and compared using the bootstrap method with 1000 resamplings. The overall incidence rates above and below each cut point were calculated. For any comorbidities that had an identifiable cut point, baseline characteristics and prevalence of any concurrent comorbidities were compared between patients who developed the comorbidity and those who did not develop the comorbidity.

All statistical analyses were conducted using R version 3.6.3 (R Foundation for Statistical Computing).

### Incidence Versus Prevalence Cut Point Analysis

Studies have identified cut points for diseases such as diabetes, hypertension, and hyperlipidemia using both incidence and prevalence [6,19]. As there is no standardized method to determine cut points, we analyzed cut point differences between prevalent and incident cases. For any comorbidities that had an identifiable cut point, we used the bootstrap method with 1000 resamplings to determine cut points and *P* values comparing incident and prevalent cases.

## Results

### Patient Characteristics

**Figure 1:**
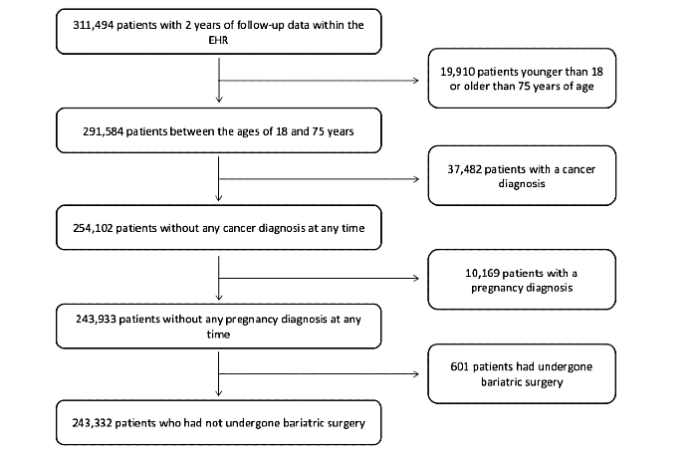
Study cohort creation (Strengthening the Reporting of Observational Studies in Epidemiology diagram). EHR: electronic health record.

Over 300,000 patients had at least three clinical visits during the study period. After applying exclusion criteria, 243,332 patients met inclusion criteria (Figure 1). The mean age was 46.8 (SD 15.3) years (Table 1). Of the patients, 54.9% (133,654/243,332) of the patients were female, and 88.7% (215,950/243,332) patients were White and non-Hispanic. The mean BMI was 29.1 (SD 7.0) kg/m^2^, and 36.8% (89,660/243,332) of patients had a BMI ≥30 kg/m^2^. In our study cohort, 57.7% (139,753/243,332) of patients had never smoked or used tobacco products, whereas 14.1% (34,328/243,332) of patients were active smokers. Hyperlipidemia and hypertension were the most common comorbidities, affecting 24.3% (59,097/243,332) patients and 21.5% (52,365/243,332) of the study population, respectively (Table 1).

**Table 1:** Baseline demographics and patient characteristics (N=243,332).

Characteristics			Values
Age (years), mean (SD)			46.8 (15.3)
**Sex, n (%)**			
	Male	109,678 (45.1)	
	Female	133,654 (54.9)	
**Race or ethnicity, n (%)**			
	White, non-Hispanic	215,950 (88.7)	
	Black, non-Hispanic	9463 (3.9)	
	Asian, non-Hispanic	6621 (2.7)	
	Native American, non-Hispanic	1161 (0.5)	
	Hispanic	7730 (3)	
	Other or unspecified	2767 (1.1)	
**Baseline BMI category (kg/m^2^)^a^, n (%)**			
	Underweight (<18.5)	3000 (1.2)	
	Normal (18.5-24.9)	72,803 (29.9)	
	Overweight (25.0-29.9)	77,869 (32)	
	Class 1 obesity (30.0-34.9)	48,213 (19.8)	
	Class 2 obesity (35.0-39.9)	23,371 (9.6)	
	Class 3 obesity (>40)	18,076 (7.4)	
**Insurance type, n (%)**			
	Commercial	191,697 (78.8)	
	Medicare	31,778 (5.7)	
	Medicaid	6032 (2.5)	
	Other or unspecified	13,825 (5.7)	
**Prevalence of comorbidities, n (%)**			
	Anxiety	33,984 (14)	
	Coronary artery disease	9543 (3.9)	
	Cerebrovascular disease	3076 (1.3)	
	Chronic pain	14,479 (6)	
	Depression	32,210 (13.2)	
	Gastroesophageal reflux	29,512 (12.1)	
	Hyperlipidemia	59,097 (24.3)	
	Hypertension	52,365 (21.5)	
	Obstructive sleep apnea	13,746 (5.6)	
	Osteoarthritis	21,408 (8.8)	
	Type 2 diabetes mellitus	18,182 (7.5)	
**Smoking status, n (%)**			
	Active smoker	34,328 (14.1)	
	Former smoker	64,331 (26.4)	
	Passive smoker	2746 (1.1)	
	Nonsmoker	139,753 (57.4)	

^a^The mean baseline BMI was 29.1 kg/m^2^ (SD 7.0 kg/m^2^).

### Incidence of 11 Comorbidities and Their Associations With BMI

The highest 1-year incidence rates were for hyperlipidemia (4.0 cases per 100 person-years) and hypertension (3.6 cases per 100 person-years; Multimedia Appendix 3 contains the full table of 1-year incidence rates). CAD and cerebrovascular disease had the lowest 1-year incidence rates (0.9 and 0.4 cases per 100-person-years, respectively).

In quantile regression, when comparing the median BMI of those who developed each comorbidity (incident group) versus the median BMI of those who did not, we found statistically significant differences in the median BMIs for all obesity-related comorbidities (Multimedia Appendix 4 contains the full table of the quantile regression analysis evaluating associations between BMI and comorbidity incidence). The median BMIs of the incident groups were higher for all comorbidities except for anxiety (–0.6 kg/m^2^; 95% CI –0.8 to –0.4).

After adjusting for age, sex, race or ethnicity, and smoking status, we found statistically significant differences in the median BMIs for all comorbidities except anxiety and cerebrovascular disease (Multimedia Appendix 4 contains the full table of the quantile regression analysis evaluating associations between BMI and comorbidity incidence). The adjusted median BMIs of the incident groups were higher for all comorbidities. The greatest differences in adjusted median BMI were for OSA (6.0 kg/m^2^; 95% CI 5.7-6.4) and T2DM (5.0 kg/m^2^; 95% CI 4.6-5.4).

### BMI Cut Points for All Study Patients

Six comorbidities had BMI cut points: CAD, hyperlipidemia, hypertension, OSA, osteoarthritis, and T2DM (Table 2). Hyperlipidemia had the lowest cut point (27.1 kg/m^2^; sensitivity=68.8%; specificity=52.1%), followed by CAD (27.7 kg/m^2^; sensitivity=66.5%; specificity=50.5%), hypertension (28.4 kg/m^2^; sensitivity=62.3%; specificity=60.7%), osteoarthritis (28.7 kg/m^2^; sensitivity=58.7%; specificity=51.7%), OSA (30.1 kg/m^2^; sensitivity=72%; specificity=66.6%), and T2DM (30.9 kg/m^2^; sensitivity=63.3%; specificity=70.9%).

**Figure 2:**
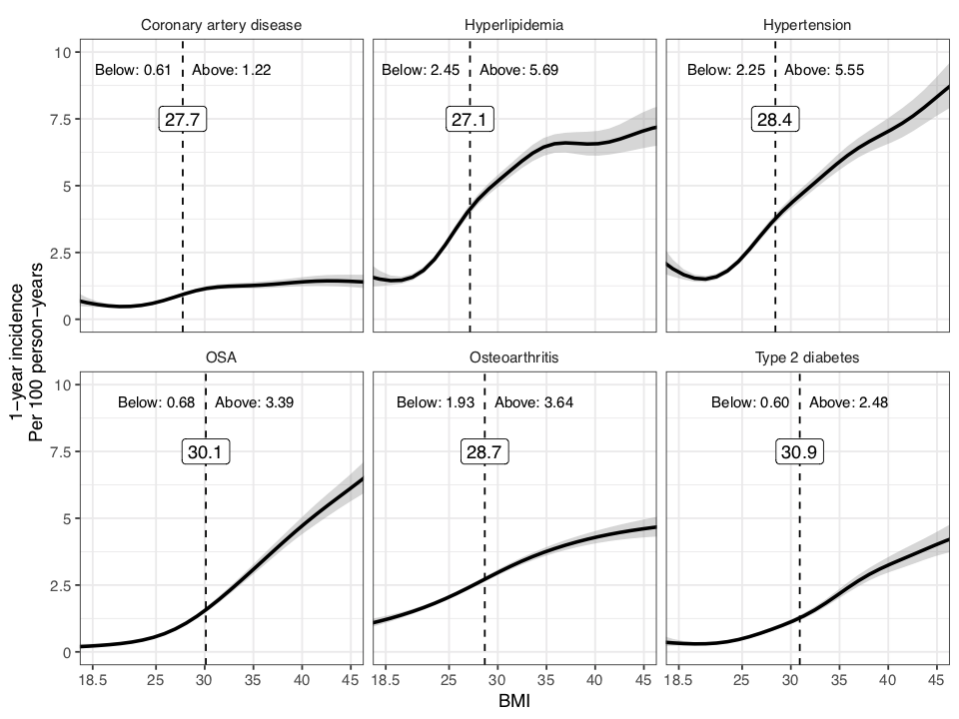
Cut points and comorbidity incidence. Gray shaded areas represent 95% CIs. The dotted line and the values in the box represent BMI cut points. "Below" corresponds to overall disease incidence (per 100 person-years) for all patients with a BMI that is less than the cut point. "Above" corresponds to overall disease incidence (per 100 person-years) for all patients with a BMI that is greater than the cut point. OSA: obstructive sleep apnea.

The 1-year incidence rates above the cut point were higher than the rates below the cut point for the six comorbidities that had identified cut points (Figure 2). The greatest differences were for OSA (0.7 cases per 100 person-years below vs 3.4 cases per 100 person-years above the cut point) and T2DM (0.6 cases per 100 person-years below vs 2.5 cases per 100 person-years above the cut point).

When comparing baseline demographics for the comorbidities with an identifiable cut point (CAD, hyperlipidemia, hypertension, OSA, osteoarthritis, and T2DM), we found that patients who developed each disease were older and more likely to be male than those who did not develop each disease for all six comorbidities (Multimedia Appendices 5-10 contain tables comparing baseline characteristics of patients who developed each comorbidity vs those who did not for all six comorbidities with a cut point). Patients who developed each comorbidity had a higher prevalence of each of the other five comorbidities with an identifiable cut point. For example, patients who developed hypertension had a higher prevalence of CAD, hyperlipidemia, OSA, osteoarthritis, and T2DM.

**Table 2:** Cut points for comorbidities.

Comorbidity	AUROC^a^	Youden index	Sensitivity, %	Specificity, %	Cut point (kg/m^2^)
Anxiety	0.477	N/A^b^	N/A	N/A	N/A
Coronary artery disease	0.603	0.170	66.5	50.5	27.7
Cerebrovascular disease	0.561	N/A	N/A	N/A	N/A
Chronic pain	0.559	N/A	N/A	N/A	N/A
Depression	0.521	N/A	N/A	N/A	N/A
Gastroesophageal reflux	0.555	N/A	N/A	N/A	N/A
Hyperlipidemia	0.637	0.209	68.8	52.1	27.1
Hypertension	0.653	0.230	62.3	60.7	28.4
Obstructive sleep apnea	0.754	0.386	72	66.6	30.1
Osteoarthritis	0.606	0.161	58.7	51.7	28.7
Type 2 diabetes mellitus	0.725	0.341	63.3	70.9	30.9

^a^AUROC: area under the receiver operating curve.

^b^N/A: not applicable.

### BMI Cut Points by Sex

Both male and female patients had cut points for hyperlipidemia, hypertension, OSA, and T2DM, but only female patients had cut points for CAD and osteoarthritis (Table 3; Multimedia Appendix 11 contains the full table of AUROC, Youden index, sensitivity, and specificity values for cut points by sex and race or ethnicity). Female patients had a statistically significant lower cut point for T2DM (29.9 vs 32.1 kg/m^2^; *P*=.02). There were no differences in other cut points between the male and female patients.

**Table 3:** Cut points by sex.

Comorbidity	Male cut point (kg/m^2^)	Female cut point (kg/m^2^)	*P* value^a^
Coronary artery disease	N/A^b^	27.8	N/A
Hyperlipidemia	28.3	28.6	.78
Hypertension	28.8	28.5	.84
Obstructive sleep apnea	31.3	30.2	.74
Osteoarthritis	N/A	29.2	N/A
Type 2 diabetes mellitus	32.1	29.9	.02

^a^*P* value indicates the comparison of cut points between male and female patients.

^b^N/A: not applicable.

### BMI Cut Points by Race or Ethnicity

When evaluating cut points by race or ethnicity, Black patients had higher cut points for hypertension (30.3 vs 28.7 kg/m^2^ for White patients; *P*<.001) and OSA (35.1 vs 30.1 kg/m^2^; *P*=.005; Table 4; Multimedia Appendix 11 contains the full table of AUROC, Youden index, sensitivity, and specificity values for cut points by sex and race or ethnicity). Asian patients had lower cut points for hyperlipidemia (24.1 vs 26.5 kg/m^2^ for White patients; *P*=.02), OSA (29.0 vs 30.1 kg/m^2^; *P*=.02), and T2DM (27.5 vs 31.3 kg/m^2^; *P*=.04). Native American patients had lower cut points for hypertension (26.0 vs 28.7 kg/m^2^ for White patients) and T2DM (29.3 vs 31.3 kg/m^2^) and a higher cut point for hyperlipidemia (28.8 vs 26.5 kg/m^2^), but these differences were not statistically significant. For Hispanic patients, we only identified a cut point for OSA (31.3 kg/m^2^; sensitivity=69.2%; specificity=70.4%).

**Table 4:** Cut points by race or ethnicity.

Comorbidity	White, non-Hispanic	Black, non-Hispanic			Asian, non-Hispanic			Native American, non-Hispanic			Hispanic	
	Cut point (kg/m^2^)	Cut point (kg/m^2^)	*P* value^a^	Cut point (kg/m^2^)		*P* value	Cut point (kg/m^2^)		*P* value	Cut point (kg/m^2^)		*P* value
Coronary artery disease	27.4	N/A^b^	N/A	N/A		N/A	N/A		N/A	N/A		N/A
Hyperlipidemia	26.5	N/A	N/A	24.1		.02	28.8		.41	N/A		N/A
Hypertension	28.7	30.3	.001	25.0		.23	26.0		.45	N/A		N/A
Obstructive sleep apnea	30.1	35.1	.005	29.0		.02	N/A		N/A	31.3		.08
Osteoarthritis	28.7	31.0	.55	N/A		N/A	N/A		N/A	N/A		N/A
Type 2 diabetes mellitus	31.3	31.3	.91	27.5		.04	29.3		.55	N/A		N/A

^a^*P* value indicates the comparison to cut points for White, non-Hispanic patients.

^b^N/A: not applicable.

### Incidence Versus Prevalence Cut Point Analysis

For the six comorbidities that had BMI cut points, we found no statistically significant differences in cut points between the incident and prevalent cases for CAD, hypertension, OSA, and osteoarthritis (Multimedia Appendix 12 contains the full table of incidence vs prevalence cut points). There were statistically significant differences between incidence and prevalence cut points for hyperlipidemia (27.5 vs 27.0 kg/m^2^; *P*=.02) and T2DM (30.7 vs 30.0 kg/m^2^; *P*<.001).

## Discussion

### Principal Findings

Our findings suggest that the BMI cut points or thresholds beyond which disease incidence can be accurately detected for developing six obesity-related comorbidities occur when patients are overweight or barely meet the criteria for class 1 obesity. The cut points for developing CAD, hyperlipidemia, hypertension, and osteoarthritis were in the overweight category, while the cut points for OSA and T2DM occurred at the transition between overweight and class 1 obesity. In our study cohort, female patients had lower cut points for T2DM. Asian patients had lower cut points for hyperlipidemia, OSA, and T2DM, while Black patients had higher cut points for hypertension and OSA.

Most cut points identified in our study were within the overweight BMI category. Published studies are currently mixed with regard to the association between being overweight and the development of *obesity-related* comorbidities. The meta-analysis by Guh et al [9] found that the relative risks for comorbidities, such as T2DM and CAD, increased when patients were overweight but increased most when patients were obese. Other studies, such as the cross-sectional study by Nguyen et al [16], which used National Health and Nutrition Examination Survey (NHANES) data, demonstrated that higher BMIs were associated with an increased risk of these diseases. In contrast, a retrospective cohort study of Swiss adults by Faeh et al [10] showed increased mortality rates in patients with obesity because of CAD but not in patients who were overweight. Despite numerous studies identifying associations between these chronic diseases and obesity, no studies have identified these cut points in multiracial or ethnic populations.

We found that female patients had a lower cut point for T2DM than male patients. The literature is inconclusive regarding the association between sex and the development of obesity-related comorbidities. The retrospective study by Chu et al [6] found lower cut points for both hypertension and T2DM in Taiwanese women than men. A large cohort study evaluating the incidence of hypertension in Japanese adults with obesity showed that the relationship between BMI and hypertension was influenced by sex, with female patients experiencing a greater risk of developing hypertension [20]. In contrast, a retrospective study by Ong et al [21] of US adults using data from NHANES showed no difference in the risk of hypertension between men and women. Although our results showed no differences in hyperlipidemia cut points between male and female patients, a retrospective cohort study by Tseng et al [19] demonstrated a lower cut point for hyperlipidemia in Taiwanese women than men.

Our study found that compared with White patients, Black patients had higher cut points for hypertension and OSA. The cross-sectional study by Fontaine et al [22] using NHANES data found that Black patients experienced obesity-related morbidity, such as reduction in lifespan, at higher BMIs than White patients. In a review, Wagner and Heyward [23] hypothesized that differences in the development of obesity-related comorbidities between Black patients and those of different racial or ethnic backgrounds stemmed from variations in body composition; Black patients typically have higher BMIs than White patients despite having similar levels of body fat.

We also found that Asian patients had lower cut points. This is supported by the Expert Committee of the World Health Organization, which concluded that Asian populations have different associations between BMI and obesity-related diseases and that the cut points of obesity-related comorbidities in Asians varied between 22.0-25.0 kg/m^2^ [4,7]. The population-based cross-sectional study by Cheong et al [24] of Malaysian adults identified BMI cut points for predicting the presence of diabetes, hypertension, and hyperlipidemia to be between 23.3-24.1 kg/m^2^ in men and 24.0-25.4 kg/m^2^ in women. A prospective study by Chan et al [25] of Chinese adults diagnosed with CAD identified a BMI cut point of 27.3 kg/m^2^ for the development of OSA. The lower cut points in Asian patients have been attributed to a multitude of genetic and metabolic differences between Asian and White patients, such as different associations between BMI and body fat percentage in Asian versus White populations [4,7]. In addition, there may be differences among the various Asian subgroups. A secondary analysis by Jih et al [7] of the California Health Interview Survey found the highest rates of overweight or obesity and diabetes in Filipino populations, suggesting that genetic, lifestyle, and dietary factors may account for variations in cut points and disease risk.

### Study Implications

Our results suggest that although some current screening guidelines incorporating BMI have appropriate cut points, others may need to be revised. For example, the United States Preventative Services Task Force (USPSTF) recommends screening for T2DM [26] and hypertension [27] at a BMI cut point of 25 kg/m^2^. Our BMI cut points of 30.9 kg/m^2^ and 28.4 kg/m^2^ for T2DM and hypertension, respectively, support these guideline cut points.

In contrast, guidelines for OSA screening vary. The American Academy of Sleep Medicine recommends OSA screening for adults with a BMI ≥30 kg/m^2^ [28]. The American Federal Aviation Administration and the US Federal Motor Carrier Safety Administration suggest that pilots with BMI ≥40 kg/m^2^ and drivers with BMI ≥35 kg/m^2^, respectively, should be screened for OSA [29,30]. The American Academy of Sleep Medicine BMI cut point of 30 kg/m^2^ and our cut point of 30.1 kg/m^2^ suggest that the Federal Aviation Administration and US Federal Motor Carrier Safety Administration screening cut points for OSA may be too high.

BMI is not included in the current screening recommendations for hyperlipidemia, CAD, or osteoarthritis. Although the USPSTF and American College of Cardiology/American Heart Association have guidelines for hyperlipidemia screening and statin use for some patients who meet age and cardiovascular disease risk criteria, BMI is not one of those criteria [31,32]. This study identified a cut point of 27.1 kg/m^2^ for hyperlipidemia risk, indicating that the inclusion of BMI as a risk factor may be warranted. The USPSTF does not recommend screening for CAD but suggests that clinicians offer or refer adults with a BMI ≥30 kg/m^2^ for behavioral weight loss therapy to prevent CAD development [33]. We are not aware of any USPSTF or professional society screening recommendations for osteoarthritis. Screening questionnaires for osteoarthritis exist [34] and could be provided to patients who exceed the BMI cut point of 28.7 kg/m^2^. We also identified sex and race or ethnicity differences that may need to be considered when screening adults for obesity-related comorbidities.

Our previous EHR publication found that our patient population was demographically similar to the US adult population [35]; thus, our findings may be generalizable to US adults. However, further investigation of the BMI cut points identified in this study using multi-institutional EHR data sets would further elucidate whether our findings are generalizable. If the BMI cut points are similar within multi-institutional EHR data sets, screening recommendations for some comorbidities may need to be re-evaluated to help guide health care providers on when to screen patients for obesity-related comorbidities.

### Limitations

First, although our methodology using the Youden index is established in the literature [6,19], there is no *gold standard* method for determining optimal cut points for continuous data, such as BMI. Some investigators have used disease prevalence rather than incidence to establish cut points. Our analysis comparing cut point calculations using incidence versus prevalence identified no clinically significant differences. We believe that cut points determined with incidence have more clinical utility because incidence evaluates the development of disease, whereas prevalence describes a disease that has already been diagnosed. Second, most Youden indices, sensitivities, and specificities were low, which suggests that BMI is not a perfect screening tool for these diseases. However, it has significant clinical use because it is recorded for most patients in the EHR, whereas other markers, such as waist circumference and biomarkers, are not. In addition, the AUROCs were >0.6, indicating that our analyses were able to discriminate between those with and without the disease. Third, there may be selection bias, given that all patients were required to have data in our EHR spanning at least 2 years. For example, our EHR had a lower percentage of Medicaid patients than the national estimates. Fourth, our study was observational, so no inferences can be made about causation. Finally, there may be inaccuracies in our data set because of errors in data entry by health care providers. We removed biologically implausible values using our BMI algorithm, but coding inaccuracies in the ICD-9 and ICD-10 entries may still exist.

### Conclusions

The BMI cut points that accurately predict the risks of developing six obesity-related comorbidities (CAD, hyperlipidemia, hypertension, OSA, osteoarthritis, and T2DM) occurred when patients were overweight or barely met the criteria for class 1 obesity. Weight loss counseling for these patients is important because they are at an increased risk of morbidity and mortality related to obesity. Further studies using longitudinal, national data are needed to determine whether screening guidelines for CAD, hyperlipidemia, OSA, and osteoarthritis should be reconsidered.

## Appendix

Multimedia Appendix 1 Strengthening the Reporting of Observational Studies in Epidemiology statement checklist.

Multimedia Appendix 2 International Classification of Disease-9 and International Classification of Disease-10 codes used to identify comorbidities.

Multimedia Appendix 3 The 1-year incidence rates of comorbidities.

Multimedia Appendix 4 Quantile regression analysis of the associations between the incidence of comorbidities and median BMIs.

Multimedia Appendix 5 Comparison of baseline characteristics between patients who developed coronary artery disease and those who did not.

Multimedia Appendix 6 Comparison of baseline characteristics between patients who developed hyperlipidemia and those who did not.

Multimedia Appendix 7 Comparison of baseline characteristics between patients who developed hypertension and those who did not.

Multimedia Appendix 8 Comparison of baseline characteristics between patients who developed obstructive sleep apnea and those who did not.

Multimedia Appendix 9 Comparison of baseline characteristics between patients who developed osteoarthritis and those who did not.

Multimedia Appendix 10 Comparison of baseline characteristics between patients who developed type 2 diabetes mellitus and those who did not.

Multimedia Appendix 11 Area under the receiving operating curve, Youden index, and sensitivity or specificity of sex and race or ethnicity-specific cut points.

Multimedia Appendix 12 Incidence versus prevalence cut points.

## Abbreviations

AUROC: area under the receiver operating curve
CAD: coronary artery disease
EHR: electronic health record
ICD: International Classification of Disease
NHANES: National Health and Nutrition Examination Survey
NIH: National Institutes of Health
OSA: obstructive sleep apnea
T2DM: type 2 diabetes mellitus
USPSTF: United States Preventative Services Task Force
