# Supplementary material for: Obesity and BMI Cut Points for Associated Comorbidities: Electronic Health Record Study
Source: J Med Internet Res. 2021 Aug 9;23(8):e24017. doi: 10.2196/24017 (PMC8386370; doi:10.2196/24017)
Supplement: Multimedia Appendix 2 [file jmir_v23i8e24017_app2.docx]

**Appendix 2.** ICD-9 and -10 Codes Used to Identify Comorbidities

| **Comorbidity** | **Code type** | **Codes** |
| --- | --- | --- |
| Anxiety | ICD-9 | 300.X |
|  | ICD-10 | F41.X, F43.0, F43.22, F43.23 |
| Coronary artery disease | ICD-9 | 410.X, 411.0, 411.1, 411.8, 411.81, 411.89, 413.0, 413.1, 413.9, 414.0, 414.00, 414.01, 414.02, 414.03, 414.04, 414.05, 414.06, 414.07, 414.2, 414.3, 414.4, 414.8, 414.9, 429.2, 429.7, 429.71, 429.79, V45.81 |
|  | ICD-10 | I20.0, I20.1, I20.8, I20.9, I21.X, I22.0, I22.1, I22.2, I22.8, I22.9, I23.0, I23.1, I23.2, I23.3, I23.4, I23.5, I23.6, I23.7, I23.8, I24.0, I24.1, I24.8, I24.9, I25.X, T82.2, T82.21 |
| Cerebrovascular disease | ICD-9 | 433.X, 434.X, 435.X, 437.0, 437.1, 437.2, 438.X |
|  | ICD-10 | I63.X, I65.X, I66.X, I67.2, I67.81, I67.82, I60.3, I69.3X |
| Chronic pain | ICD-9 | 338.2, 338.29, 338.4 |
|  | ICD-10 | F45.4, F45.41, F45.42, G89.2, G89.29, G89.4 |
| Depression | ICD-9 | 300.4, 309.0, 309.1, 311 |
|  | ICD-10 | F32.X, F33.X, F34.X, F39.X, F43.21, F43.23 |
| Gastroesophageal reflux | ICD-9 | 530.1, 530.10, 530.11, 530.12, 530.13, 530.19, 530.8, 530.81, 530.85, 787.1 |
|  | ICD-10 | K20.0, K20.8, K20.9, K21.0, K21.9, K22.7, K22.70, K22.71, K22.710, K22.711, K22.719, R12 |
| Hyperlipidemia | ICD-9 | 272.0, 272.1, 272.2, 272.3, 272.4 |
|  | ICD-10 | E78.0, E78.00, E78.01, E78.1, E78.2, E78.3, E78.4, E78.5 |
| Hypertension | ICD-9 | 401.X, 402.X, 403.X, 404.X |
|  | ICD-10 | I10, I11.X, I12.X, I13.X, I16.X |
| Obstructive sleep apnea | ICD-9 | 327.2X, 780.51, 780.53, 780.57, 786.03 |
|  | ICD-10 | G47.3X, R06.81 |
| Osteoarthritis | ICD-9 | 715.X |
|  | ICD-10 | M15.X, M16.X, M17.X, M18.X, M19.X |
| Type 2 diabetes mellitus | ICD-9 | 250.X |
|  | ICD-10 | E11.X |

“X” represents any possible number
