# Supplementary material for: Obesity and BMI Cut Points for Associated Comorbidities: Electronic Health Record Study
Source: J Med Internet Res. 2021 Aug 9;23(8):e24017. doi: 10.2196/24017 (PMC8386370; doi:10.2196/24017)
Supplement: Multimedia Appendix 3 [file jmir_v23i8e24017_app3.docx]

**Appendix 3.** One-Year Incidence Rates of Comorbidities

| **Comorbidity** | **Total at risk** | **Number of new cases during 1-year interval** | **1-year incidence rate (per 100 person-years)** |
| --- | --- | --- | --- |
| Anxiety | 209,348 | 6,762 | 3.23 |
| Coronary artery disease | 233,789 | 2,134 | 0.91 |
| Cerebrovascular disease | 240,256 | 878 | 0.37 |
| Chronic pain | 228,853 | 6,481 | 2.83 |
| Depression | 211,122 | 6,231 | 2.95 |
| Gastroesophageal reflux | 213,820 | 5,912 | 2.76 |
| Hyperlipidemia | 184,235 | 7,422 | 4.03 |
| Hypertension | 190,967 | 6,827 | 3.57 |
| Obstructive sleep apnea | 229,586 | 3,672 | 1.60 |
| Osteoarthritis | 221,924 | 5,917 | 2.67 |
| Type 2 diabetes mellitus | 225,150 | 2,603 | 1.16 |
