# Supplementary material for: Obesity and BMI Cut Points for Associated Comorbidities: Electronic Health Record Study
Source: J Med Internet Res. 2021 Aug 9;23(8):e24017. doi: 10.2196/24017 (PMC8386370; doi:10.2196/24017)
Supplement: Multimedia Appendix 4 [file jmir_v23i8e24017_app4.docx]

**Appendix 4.** Quantile Regression Analysis of Associations Between Incidence of Comorbidities and Median BMI

|  | **Unadjusted** | **Adjusted** |
| --- | --- | --- |
| **Comorbidity** | **Difference in median BMI (kg/m^2^) [95% confidence interval]** | **Difference in median BMI (kg/m^2^) [95% confidence interval]** |
| Anxiety | -0.56 [-0.76, -0.35] | -0.09 [-0.30, 0.11] |
| Coronary artery disease | 2.44 [2.16, 2.72] | 0.87 [0.54, 1.21] |
| Cerebrovascular disease | 1.40 [1.03, 1.78] | 0.18 [-0.30, 0.65] |
| Chronic pain | 1.42 [1.20, 1.64] | 1.01 [0.83, 1.20] |
| Depression | 0.49 [0.29, 0.70] | 0.74 [0.53, 0.94] |
| Gastroesophageal reflux | 1.37 [1.15, 1.60] | 0.95 [0.73, 1.16] |
| Hyperlipidemia | 3.16 [2.97, 3.34] | 2.24 [2.06, 2.43] |
| Hypertension | 3.58 [3.41, 3.76] | 2.60 [2.40, 2.81] |
| Obstructive sleep apnea | 6.74 [6.46, 7.03] | 6.00 [5.66, 6.35] |
| Osteoarthritis | 2.51 [2.31, 2.72] | 1.56 [1.33, 1.79] |
| Type 2 diabetes mellitus | 6.08 [5.67, 6.49] | 5.01 [4.62, 5.39] |
