# Supplementary material for: Obesity and BMI Cut Points for Associated Comorbidities: Electronic Health Record Study
Source: J Med Internet Res. 2021 Aug 9;23(8):e24017. doi: 10.2196/24017 (PMC8386370; doi:10.2196/24017)
Supplement: Multimedia Appendix 5 [file jmir_v23i8e24017_app5.docx]

**Appendix 5.** Comparison of Baseline Characteristics Between Patients Who Developed Coronary Artery Disease Versus Those Who Did Not

|  | **Patients who developed coronary artery disease**  **(n = 2,134 patients)** | **Patients who did not develop coronary artery disease**  **(n = 231,655 patients)** |
| --- | --- | --- |
| **Age, mean (SD) (years)** | 60.8 (9.6) | 46.0 (15.2) |
| **Sex (n,%)** |  |  |
| Male | 1,353 (63.0) | 101,450 (44.0) |
| Female | 781 (37.0) | 130,205 (56.0) |
| **Race/ethnicity (n,%)** |  |  |
| White, non-Hispanic | 1,901 (89.1) | 205,341 (88.6) |
| Black, non-Hispanic | 116 (5.4) | 8,964 (3.9) |
| Asian, non-Hispanic | 29 (1.4) | 6,444 (2.8) |
| Native American, non-Hispanic | 15 (0.7) | 1,089 (0.5) |
| Hispanic | 58 (2.7) | 7,132 (3.1) |
| Other/unspecified | 15 (0.7) | 2,685 (1.2) |
| **Baseline BMI category (n,%)** |  |  |
| Underweight (BMI < 18.5 kg/m^2^) | 28 (1.3) | 2,917 (1.3) |
| Normal (18.5 – 24.9 kg/m^2^) | 350 (16.4) | 71,118 (30.7) |
| Overweight (25.0 – 29.9 kg/m^2^) | 676 (31.7) | 74,137 (32.0) |
| Class 1 obesity (30.0 – 34.9 kg/m^2^) | 554 (26.0) | 44,968 (19.4) |
| Class 2 obesity (35.0 – 39.9 kg/m^2^) | 279 (13.1) | 21,689 (9.4) |
| Class 3 obesity (> 40 kg/m^2^) | 247 (11.6) | 16,826 (7.3) |
| **Insurance type (n,%)** |  |  |
| Commercial | 1,173 (55.0) | 185,214 (80.0) |
| Medicare | 800 (37.5) | 27,442 (11.8) |
| Medicaid | 73 (3.4) | 5,697 (2.5) |
| Other/unspecified | 88 (4.1) | 13,302 (5.7) |
| **Prevalence of comorbidities (n,%)** |  |  |
| Anxiety | 197 (9.0) | 32,751 (14.0) |
| Coronary artery disease | -- | -- |
| Cerebrovascular disease | 89 (4.0) | 2,301 (1.0) |
| Chronic pain | 200 (9.0) | 13,497 (6.0) |
| Depression | 271 (13.0) | 30,692 (13.0) |
| Gastroesophageal reflux | 288 (13.0) | 27,546 (12.0) |
| Hyperlipidemia | 805 (38.0) | 51,702 (22.0) |
| Hypertension | 918 (43.0) | 45,375 (20.0) |
| Obstructive sleep apnea | 204 (10.0) | 12,154 (5.0) |
| Osteoarthritis | 322 (15.0) | 19,537 (8.0) |
| Type 2 diabetes mellitus | 536 (25.0) | 14,870 (6.0) |
| **Smoking status (n,%)** |  |  |
| Active smoker | 444 (20.8) | 32,326 (14.0) |
| Former smoker | 865 (40.5) | 58,856 (25.4) |
| Passive smoker | 12 (0.6) | 2,681 (1.2) |
| Never smoker | 778 (36.5) | 135,763 (58.6) |
