# Supplementary material for: Obesity and BMI Cut Points for Associated Comorbidities: Electronic Health Record Study
Source: J Med Internet Res. 2021 Aug 9;23(8):e24017. doi: 10.2196/24017 (PMC8386370; doi:10.2196/24017)
Supplement: Multimedia Appendix 6 [file jmir_v23i8e24017_app6.docx]

**Appendix 6.** Comparison of Baseline Characteristics Between Patients Who Developed Hyperlipidemia Versus Those Who Did Not

|  | **Patients who developed hyperlipidemia**  **(n = 7,422 patients)** | **Patients who did not develop hyperlipidemia**  **(n = 176,813 patients)** |
| --- | --- | --- |
| **Age, mean (SD) (years)** | 54.0 (12.7) | 43.2 (15.0) |
| **Sex (n,%)** |  |  |
| Male | 3,990 (53.8) | 73,249 (41.0) |
| Female | 3,432 (46.2) | 103,564 (59.0) |
| **Race/ethnicity (n,%)** |  |  |
| White, non-Hispanic | 6,530 (88.0) | 155,419 (87.9) |
| Black, non-Hispanic | 337 (4.5) | 7,617 (4.3) |
| Asian, non-Hispanic | 234 (3.2) | 5,025 (2.8) |
| Native American, non-Hispanic | 35 (0.5) | 878 (0.5) |
| Hispanic | 214 (2.9) | 5,794 (3.3) |
| Other/unspecified | 72 (1.0) | 2,080 (1.2) |
| **Baseline BMI category (n,%)** |  |  |
| Underweight (BMI < 18.5 kg/m^2^) | 51 (0.7) | 2,780 (1.6) |
| Normal (18.5 – 24.9 kg/m^2^) | 1,288 (17.4) | 62,719 (35.5) |
| Overweight (25.0 – 29.9 kg/m^2^) | 2,381 (32.1) | 55,602 (31.4) |
| Class 1 obesity (30.0 – 34.9 kg/m^2^) | 1,883 (25.4) | 30,489 (17.2) |
| Class 2 obesity (35.0 – 39.9 kg/m^2^) | 1,002 (13.5) | 14,145 (8.0) |
| Class 3 obesity (> 40 kg/m^2^) | 817 (11.0) | 11,078 (6.3) |
| **Insurance type (n,%)** |  |  |
| Commercial | 5,100 (68.7) | 143,741 (81.3) |
| Medicare | 1,753 (23.6) | 16,811 (9.5) |
| Medicaid | 189 (2.5) | 4,964 (2.8) |
| Other/unspecified | 380 (5.1) | 11,297 (6.4) |
| **Prevalence of comorbidities (n,%)** |  |  |
| Anxiety | 825 (11.0) | 23,582 (13.0) |
| Coronary artery disease | 592 (8.0) | 2,361 (1.0) |
| Cerebrovascular disease | 171 (2.0) | 1,164 (1.0) |
| Chronic pain | 492 (7.0) | 9,166 (5.0) |
| Depression | 878 (12.0) | 21,152 (12.0) |
| Gastroesophageal reflux | 881 (12.0) | 16,434 (9.0) |
| Hyperlipidemia | -- | -- |
| Hypertension | 2,251 (30.0) | 18,941 (11.0) |
| Obstructive sleep apnea | 452 (6.0) | 6,675 (4.0) |
| Osteoarthritis | 734 (10.0) | 11,114 (6.0) |
| Type 2 diabetes mellitus | 996 (13.0) | 4,910 (3.0) |
| **Smoking status (n,%)** |  |  |
| Active smoker | 1,100 (14.8) | 25,627 (14.5) |
| Former smoker | 2,490 (33.6) | 40,984 (23.2) |
| Passive smoker | 60 (0.8) | 2,252 (1.3) |
| Never smoker | 3,674 (49.5) | 105,960 (59.9) |
