# Supplementary material for: Obesity and BMI Cut Points for Associated Comorbidities: Electronic Health Record Study
Source: J Med Internet Res. 2021 Aug 9;23(8):e24017. doi: 10.2196/24017 (PMC8386370; doi:10.2196/24017)
Supplement: Multimedia Appendix 7 [file jmir_v23i8e24017_app7.docx]

**Appendix 7.** Comparison of Baseline Characteristics Between Patients Who Developed Hypertension Versus Those Who Did Not

|  | **Patients who developed hypertension**  **(n = 6,827 patients)** | **Patients who did not develop hypertension**  **(n = 184,140 patients)** |
| --- | --- | --- |
| **Age, mean (SD) (years)** | 55.0 (12.5) | 43.5 (14.9) |
| **Sex (n,%)** |  |  |
| Male | 3,641 (53.3) | 77,677 (42.0) |
| Female | 3,186 (46.7) | 106,463 (58.0) |
| **Race/ethnicity (n,%)** |  |  |
| White, non-Hispanic | 6,001 (87.9) | 163,238 (88.7) |
| Black, non-Hispanic | 392 (5.7) | 6,397 (3.5) |
| Asian, non-Hispanic | 141 (2.1) | 5,358 (2.9) |
| Native American, non-Hispanic | 46 (0.7) | 861 (0.5) |
| Hispanic | 179 (2.6) | 6,077 (3.3) |
| Other/unspecified | 68 (1.0) | 2,209 (1.2) |
| **Baseline BMI category (n,%)** |  |  |
| Underweight (BMI < 18.5 kg/m^2^) | 65 (1.0) | 2,723 (1.5) |
| Normal (18.5 – 24.9 kg/m^2^) | 1,154 (16.9) | 64,846 (35.2) |
| Overweight (25.0 – 29.9 kg/m^2^) | 2,032 (29.8) | 60,672 (32.9) |
| Class 1 obesity (30.0 – 34.9 kg/m^2^) | 1,750 (25.6) | 32,451 (17.6) |
| Class 2 obesity (35.0 – 39.9 kg/m^2^) | 946 (13.9) | 13,966 (7.6) |
| Class 3 obesity (> 40 kg/m^2^) | 880 (12.9) | 9,482 (5.1) |
| **Insurance type (n,%)** |  |  |
| Commercial | 4,384 (64.2) | 151,537 (82.3) |
| Medicare | 1,843 (27.0) | 16,816 (9.1) |
| Medicaid | 234 (3.4) | 4,669 (2.5) |
| Other/unspecified | 366 (5.4) | 11,118 (6.0) |
| **Prevalence of comorbidities (n,%)** |  |  |
| Anxiety | 714 (10.0) | 25,329 (14.0) |
| Coronary artery disease | 596 (9.0) | 2,875 (2.0) |
| Cerebrovascular disease | 152 (2.0) | 1,182 (1.0) |
| Chronic pain | 471 (7.0) | 9,473 (5.0) |
| Depression | 688 (10.0) | 23,026 (13.0) |
| Gastroesophageal reflux | 766 (11.0) | 18,816 (10.0) |
| Hyperlipidemia | 1,489 (22.0) | 26,435 (14.0) |
| Hypertension | -- | -- |
| Obstructive sleep apnea | 406 (6.0) | 6,710 (4.0) |
| Osteoarthritis | 748 (11.0) | 11,911 (6.0) |
| Type 2 diabetes mellitus | 914 (13.0) | 5,251 (3.0) |
| **Smoking status (n,%)** |  |  |
| Active smoker | 1,101 (16.1) | 25,560 (13.9) |
| Former smoker | 2,327 (34.1) | 43,007 (23.4) |
| Passive smoker | 54 (0.8) | 2,295 (1.3) |
| Never smoker | 3,234 (47.4) | 111,373 (60.5) |
