# Supplementary material for: Obesity and BMI Cut Points for Associated Comorbidities: Electronic Health Record Study
Source: J Med Internet Res. 2021 Aug 9;23(8):e24017. doi: 10.2196/24017 (PMC8386370; doi:10.2196/24017)
Supplement: Multimedia Appendix 8 [file jmir_v23i8e24017_app8.docx]

**Appendix 8.** Comparison of Baseline Characteristics Between Patients Who Developed Obstructive Sleep Apnea Versus Those Who Did Not

|  | **Patients who developed obstructive sleep apnea**  **( n = 3,672 patients)** | **Patients who did not develop obstructive sleep apnea**  **(n = 225,914 patients)** |
| --- | --- | --- |
| **Age, mean (SD) (years)** | 52.8 (12.7) | 46.3 (15.4) |
| **Sex (n,%)** |  |  |
| Male | 2,221 (60.0) | 98,507 (44.0) |
| Female | 1,451 (40.0) | 127,407 (56.0) |
| **Race/ethnicity (n,%)** |  |  |
| White, non-Hispanic | 3,298 (89.8) | 200,205 (88.6) |
| Black, non-Hispanic | 169 (4.6) | 8,732 (3.9) |
| Asian, non-Hispanic | 52 (1.4) | 6,378 (2.8) |
| Native American, non-Hispanic | 18 (0.5) | 1,078 (0.5) |
| Hispanic | 104 (2.8) | 6,951 (3.1) |
| Other/unspecified | 31 (0.8) | 2,570 (1.1) |
| **Baseline BMI category (n,%)** |  |  |
| Underweight (BMI < 18.5 kg/m^2^) | 8 (0.2) | 2,959 (1.3) |
| Normal (18.5 – 24.9 kg/m^2^) | 278 (7.6) | 71,737 (31.8) |
| Overweight (25.0 – 29.9 kg/m^2^) | 732 (19.9) | 74,412 (32.9) |
| Class 1 obesity (30.0 – 34.9 kg/m^2^) | 970 (26.4) | 43,608 (19.3) |
| Class 2 obesity (35.0 – 39.9 kg/m^2^) | 787 (21.4) | 19,727 (8.7) |
| Class 3 obesity (> 40 kg/m^2^) | 897 (24.4) | 13,471 (6.0) |
| **Insurance type (n,%)** |  |  |
| Commercial | 2,721 (74.1) | 178,524 (79.0) |
| Medicare | 712 (19.4) | 28,625 (12.7) |
| Medicaid | 86 (2.3) | 5,665 (2.5) |
| Other/unspecified | 153 (4.2) | 13,100 (5.8) |
| **Prevalence of comorbidities (n,%)** |  |  |
| Anxiety | 606 (17.0) | 30,577 (14.0) |
| Coronary artery disease | 316 (9.0) | 7,839 (3.0) |
| Cerebrovascular disease | 99 (3.0) | 2,635 (1.0) |
| Chronic pain | 366 (10.0) | 12,541 (6.0) |
| Depression | 675 (18.0) | 28,083 (12.0) |
| Gastroesophageal reflux | 694 (19.0) | 25,558 (11.0) |
| Hyperlipidemia | 1,417 (39.0) | 51,061 (23.0) |
| Hypertension | 1,449 (39.0) | 44,286 (20.0) |
| Obstructive sleep apnea | -- | -- |
| Osteoarthritis | 578 (16.0) | 18,223 (8.0) |
| Type 2 diabetes mellitus | 646 (18.0) | 14,625 (6.0) |
| **Smoking status (n,%)** |  |  |
| Active smoker | 512.0 (13.9) | 32,181 (14.2) |
| Former smoker | 1,285.0 (35.0) | 58,037 (25.7) |
| Passive smoker | 40.0 (1.1) | 2,596 (1.2) |
| Never smoker | 1,798.0 (49.0) | 130,976 (58.0) |
