# Supplementary material for: Obesity and BMI Cut Points for Associated Comorbidities: Electronic Health Record Study
Source: J Med Internet Res. 2021 Aug 9;23(8):e24017. doi: 10.2196/24017 (PMC8386370; doi:10.2196/24017)
Supplement: Multimedia Appendix 9 [file jmir_v23i8e24017_app9.docx]

**Appendix 9.** Comparison of Baseline Characteristics Between Patients Who Developed Osteoarthritis Versus Those Who Did Not

|  | **Patients who developed osteoarthritis**  **(n = 5,917 patients)** | **Patients who did not develop osteoarthritis**  **(n = 216,007 patients)** |
| --- | --- | --- |
| **Age, mean (SD) (years)** | 56.6 (11.2) | 45.3 (15.2) |
| **Sex (n,%)** |  |  |
| Male | 2,501 (42.0) | 97,829 (45.3) |
| Female | 3,416 (58.0) | 118,178 (54.7) |
| **Race/ethnicity (n,%)** |  |  |
| White, non-Hispanic | 5,400 (91.3) | 190,876 (88.4) |
| Black, non-Hispanic | 255 (4.3) | 8,410 (3.9) |
| Asian, non-Hispanic | 71 (1.2) | 6,306 (2.9) |
| Native American, non-Hispanic | 22 (0.4) | 1,037 (0.5) |
| Hispanic | 132 (2.2) | 6,844 (3.2) |
| Other/unspecified | 37 (0.6) | 2,534 (1.2) |
| **Baseline BMI category (n,%)** |  |  |
| Underweight (BMI < 18.5 kg/m^2^) | 45 (0.8) | 2,856 (1.3) |
| Normal (18.5 – 24.9 kg/m^2^) | 1,118 (18.9) | 68,110 (31.5) |
| Overweight (25.0 – 29.9 kg/m^2^) | 1,800 (30.4) | 69,640 (32.2) |
| Class 1 obesity (30.0 – 34.9 kg/m^2^) | 1,465 (24.8) | 41,443 (19.2) |
| Class 2 obesity (35.0 – 39.9 kg/m^2^) | 797 (13.5) | 19,378 (9.0) |
| Class 3 obesity (> 40 kg/m^2^) | 692 (11.7) | 14,580 (6.8) |
| **Insurance type (n,%)** |  |  |
| Commercial | 4,244 (71.7) | 172,894 (80.0) |
| Medicare | 1,271 (21.5) | 24,828 (11.5) |
| Medicaid | 118 (2.0) | 5,525 (2.6) |
| Other/unspecified | 284 (4.8) | 12,760 (5.9) |
| **Prevalence of comorbidities (n,%)** |  |  |
| Anxiety | 864 (15.0) | 29,653 (14.0) |
| Coronary artery disease | 387 (7.0) | 7,607 (4.0) |
| Cerebrovascular disease | 119 (2.0) | 2,475 (1.0) |
| Chronic pain | 709 (12.0) | 9,959 (5.0) |
| Depression | 955 (16.0) | 27,241 (13.0) |
| Gastroesophageal reflux | 1,060 (18.0) | 23,767 (11.0) |
| Hyperlipidemia | 2,131 (36.0) | 47,406 (22.0) |
| Hypertension | 2,013 (34.0) | 41,603 (19.0) |
| Obstructive sleep apnea | 573 (10.0) | 10,566 (5.0) |
| Osteoarthritis | -- | -- |
| Type 2 diabetes mellitus | 782 (13.0) | 14,444 (7.0) |
| **Smoking status (n,%)** |  |  |
| Active smoker | 758 (12.8) | 30,955 (14.3) |
| Former smoker | 2,093 (35.4) | 54,515 (25.2) |
| Passive smoker | 51 (0.9) | 2,564 (1.2) |
| Never smoker | 2,976 (50.3) | 125,853 (58.3) |
