# Supplementary material for: Obesity and BMI Cut Points for Associated Comorbidities: Electronic Health Record Study
Source: J Med Internet Res. 2021 Aug 9;23(8):e24017. doi: 10.2196/24017 (PMC8386370; doi:10.2196/24017)
Supplement: Multimedia Appendix 10 [file jmir_v23i8e24017_app10.docx]

**Appendix 10.** Comparison of Baseline Characteristics Between Patients Who Developed Type 2 Diabetes Mellitus Versus Those Who Did Not

|  | **Patients who developed type 2 diabetes mellitus**  **(n = 2,603 patients)** | **Patients who did not develop type 2 diabetes mellitus**  **(n = 222,547 patients)** |
| --- | --- | --- |
| **Age, mean (SD) (years)** | 55.6 (12.3) | 45.8 (15.2) |
| **Sex (n,%)** |  |  |
| Male | 1,430 (54.9) | 98,273 (44.0) |
| Female | 1,173 (45.1) | 124,274 (56.0) |
| **Race/ethnicity (n,%)** |  |  |
| White, non-Hispanic | 2,173 (83.5) | 198,585 (89.2) |
| Black, non-Hispanic | 205 (7.9) | 7,975 (3.6) |
| Asian, non-Hispanic | 74 (2.8) | 5,952 (2.7) |
| Native American, non-Hispanic | 23 (0.9) | 987 (0.4) |
| Hispanic | 105 (4.0) | 6,470 (2.9) |
| Other/unspecified | 23 (0.9) | 2,578 (1.2) |
| **Baseline BMI category (n,%)** |  |  |
| Underweight (BMI < 18.5 kg/m^2^) | 12.0 (0.5) | 2,950 (1.3) |
| Normal (18.5 – 24.9 kg/m^2^) | 254.0 (9.8) | 71,125 (32.0) |
| Overweight (25.0 – 29.9 kg/m^2^) | 571.0 (21.9) | 73,292 (32.9) |
| Class 1 obesity (30.0 – 34.9 kg/m^2^) | 671.0 (25.8) | 42,567 (19.1) |
| Class 2 obesity (35.0 – 39.9 kg/m^2^) | 520.0 (20.0) | 19,163 (8.6) |
| Class 3 obesity (> 40 kg/m^2^) | 575.0 (22.1) | 13,450 (6.0) |
| **Insurance type (n,%)** |  |  |
| Commercial | 1,567 (60.2) | 178,888 (80.4) |
| Medicare | 783 (30.1) | 25,610 (11.5) |
| Medicaid | 114 (4.4) | 5,275 (2.4) |
| Other/unspecified | 139 (5.3) | 12,774 (5.7) |
| **Prevalence of comorbidities (n,%)** |  |  |
| Anxiety | 254 (10.0) | 31,458 (14.0) |
| Coronary artery disease | 252 (10.0) | 6,515 (3.0) |
| Cerebrovascular disease | 78 (3.0) | 2,302 (1.0) |
| Chronic pain | 183 (7.0) | 12,539 (6.0) |
| Depression | 309 (12.0) | 28,652 (13.0) |
| Gastroesophageal reflux | 312 (12.0) | 26,036 (12.0) |
| Hyperlipidemia | 769 (30.0) | 46,052 (21.0) |
| Hypertension | 900 (35.0) | 39,448 (18.0) |
| Obstructive sleep apnea | 247 (9.0) | 10,588 (5.0) |
| Osteoarthritis | 273 (10.0) | 18,179 (8.0) |
| Type 2 diabetes mellitus | -- | -- |
| **Smoking status (n,%)** |  |  |
| Active smoker | 430 (16.5) | 31,142 (14.0) |
| Former smoker | 908 (34.9) | 56,414 (25.4) |
| Passive smoker | 23 (0.9) | 2,567 (1.2) |
| Never smoker | 1,180 (45.3) | 130,385 (58.6) |
