# Supplementary material for: Obesity and BMI Cut Points for Associated Comorbidities: Electronic Health Record Study
Source: J Med Internet Res. 2021 Aug 9;23(8):e24017. doi: 10.2196/24017 (PMC8386370; doi:10.2196/24017)
Supplement: Multimedia Appendix 11 [file jmir_v23i8e24017_app11.docx]

**Appendix 11.** AUROC, Youden’s Index, and Sensitivity/Specificity of Sex and Race/Ethnicity Specific Cut-Points

|  | **Male** | | **Female** | | **White, non-Hispanic** | | **Black, non-Hispanic** | | **Asian, non-Hispanic** | | **Native American, non-Hispanic** | | **Hispanic** | |
| --- | --- | --- | --- | --- | --- | --- | --- | --- | --- | --- | --- | --- | --- | --- |
| **Comorbidity** | **AUROC** | **Youden’s index (SN; SP)** | **AUROC** | **Youden’s index (SN; SP)** | **AUROC** | **Youden’s index (SN; SP)** | **AUROC** | **Youden’s index (SN; SP)** | **AUROC** | **Youden’s index (SN; SP)** | **AUROC** | **Youden’s index (SN; SP)** | **AUROC** | **Youden’s index (SN; SP)** |
| Anxiety | 0.472 | -- | 0.490 | -- | 0.471 | -- | 0.513 | -- | 0.532 | -- | 0.514 | -- | 0.470 | -- |
| Coronary artery disease | 0.584 | -- | 0.612 | 0.191 (64.4; 55.0) | 0.605 | 0.175 (69.2; 48.3) | 0.550 | -- | 0.561 | -- | 0.548 | -- | 0.576 | -- |
| Cerebrovascular disease | 0.539 | -- | 0.574 | -- | 0.563 | -- | 0.494 | -- | 0.433 | -- | 0.494 | -- | 0.532 | -- |
| Chronic pain | 0.530 | -- | 0.580 | -- | 0.557 | -- | 0.546 | -- | 0.519 | -- | 0.492 | -- | 0.543 | -- |
| Depression | 0.493 | -- | 0.545 | -- | 0.519 | -- | 0.508 | -- | 0.509 | -- | 0.588 | -- | 0.500 | -- |
| Gastroesophageal reflux | 0.530 | -- | 0.575 | -- | 0.558 | -- | 0.529 | -- | 0.523 | -- | 0.545 | -- | 0.531 | -- |
| Hyperlipidemia | 0.620 | 0.179 (62.2; 56.7) | 0.644 | 0.222 (58.8; 63.4) | 0.642 | 0.217 (73.6; 48.1) | 0.597 | -- | 0.628 | 0.200 (72.7; 47.4) | 0.622 | 0.344 (80.0; 54.4) | 0.571 | -- |
| Hypertension | 0.631 | 0.192 (59.3; 60.0) | 0.667 | 0.262 (62.1; 64.1) | 0.656 | 0.236 (60.9; 62.7) | 0.607 | 0.161 (58.4; 57.7) | 0.625 | 0.217 (66.7; 55.0) | 0.606 | 0.205 (82.6; 37.9) | 0.589 | -- |
| Obstructive sleep apnea | 0.733 | 0.353 (62.5; 72.8) | 0.779 | 0.443 (76.7; 67.5) | 0.751 | 0.386 (71.5; 66.8) | 0.673 | 0.435 (67.5; 76.1) | 0.693 | 0.439 (65.5; 80.5) | 0.580 | -- | 0.687 | 0.396 (69.2; 70.4) |
| Osteoarthritis | 0.589 | -- | 0.618 | 0.185 (54.7; 63.8) | 0.604 | 0.158 (58.3; 57.5) | 0.613 | 0.189 (62.0; 57.0) | 0.572 | -- | 0.413 | -- | 0.551 | -- |
| Type 2 diabetes mellitus | 0.693 | 0.299 (56.4; 76.6) | 0.755 | 0.402 (73.1; 67.1) | 0.734 | 0.357 (63.0; 72.7) | 0.650 | 0.285 (67.8; 60.7) | 0.697 | 0.321 (59.5; 72.6) | 0.625 | 0.220 (65.2; 56.7) | 0.587 | -- |

AUROC = area under the receiver operating curve; SN = sensitivity; SP = specificity
