# Supplementary material for: Obesity and BMI Cut Points for Associated Comorbidities: Electronic Health Record Study
Source: J Med Internet Res. 2021 Aug 9;23(8):e24017. doi: 10.2196/24017 (PMC8386370; doi:10.2196/24017)
Supplement: Multimedia Appendix 12 [file jmir_v23i8e24017_app12.docx]

**Appendix 12.** Incidence versus Prevalence Cut-Points

| **Comorbidity** | **Cut-point by *incidence* (kg/m^2^) [95% confidence interval]** | **Cut-point by *prevalence* (kg/m^2^) [95% confidence interval]** | ***P*-value** |
| --- | --- | --- | --- |
| Coronary artery disease | 28.0 [27.2, 28.5] | 27.7 [26.8, 28.0] | .145 |
| Hyperlipidemia | 27.5 [26.4, 28.6] | 27.0 [26.6, 27.6] | .018 |
| Hypertension | 28.1 [27.6, 29.0] | 28.2 [27.9, 28.7] | .087 |
| Obstructive sleep apnea | 30.4 [29.3, 31.4] | 30.5 [29.3, 30.9] | .654 |
| Osteoarthritis | 28.5 [27.4, 29.2] | 28.3 [27.6, 29.0] | .835 |
| Type 2 diabetes mellitus | 30.7 [29.3, 31.6] | 30.0 [29.5, 30.3] | <.001 |
